# Flavonoids as potential anti-asthmatic agents

**DOI:** 10.3389/fimmu.2026.1880181

**Published:** 2026-07-30

**Authors:** Zhuofen Deng, Haiyi Xin, Dongxin Yang, Zhenzhen Kang, Jiaying Liang, Jinhong He, Jun Gong

**Affiliations:** 1 Central Laboratory of YunFu People’s Hospital, YunFu Key Laboratory of Brain Diseases Research, Guangdong Yunfu, China; 2 Department of Pharmacy, YunFu People’s Hospital, Guangdong Yunfu, China; 3 Department of Gynecology, YunFu People’s Hospital, Guangdong Yunfu, China; 4 School of Traditional Chinese Medicine, Guangdong Pharmaceutical University, Guangdong Guangzhou, China

**Keywords:** airway remodeling, asthma, flavonoids, inflammation, T cell

## Abstract

Asthma affects approximately 260 million people globally and causes an estimated 450,000 deaths annually, posing a serious threat to human health and life quality. Current mainstay pharmacotherapies are effective for symptom control but are associated with well-documented systemic side effects and do not offer a cure. Flavonoids have garnered increasing scientific interest due to their pleiotropic biological activities; however, there is still a lack of summary on the mechanism of flavonoids in key pathological processes of asthma. This review summarizes the evidence on how flavonoids modulate key asthma pathologies, covering airway inflammation, T cell subset imbalance, airway remodeling, and smooth muscle contractility. However, clinical translation is severely constrained by poor solubility, rapid metabolism, and low oral bioavailability. Flavonoids exhibit multi-target therapeutic potential for asthma, but well-designed, large-scale clinical trials are necessary to validate these preclinical observations.

## 1 Introduction

**Figure 1:**
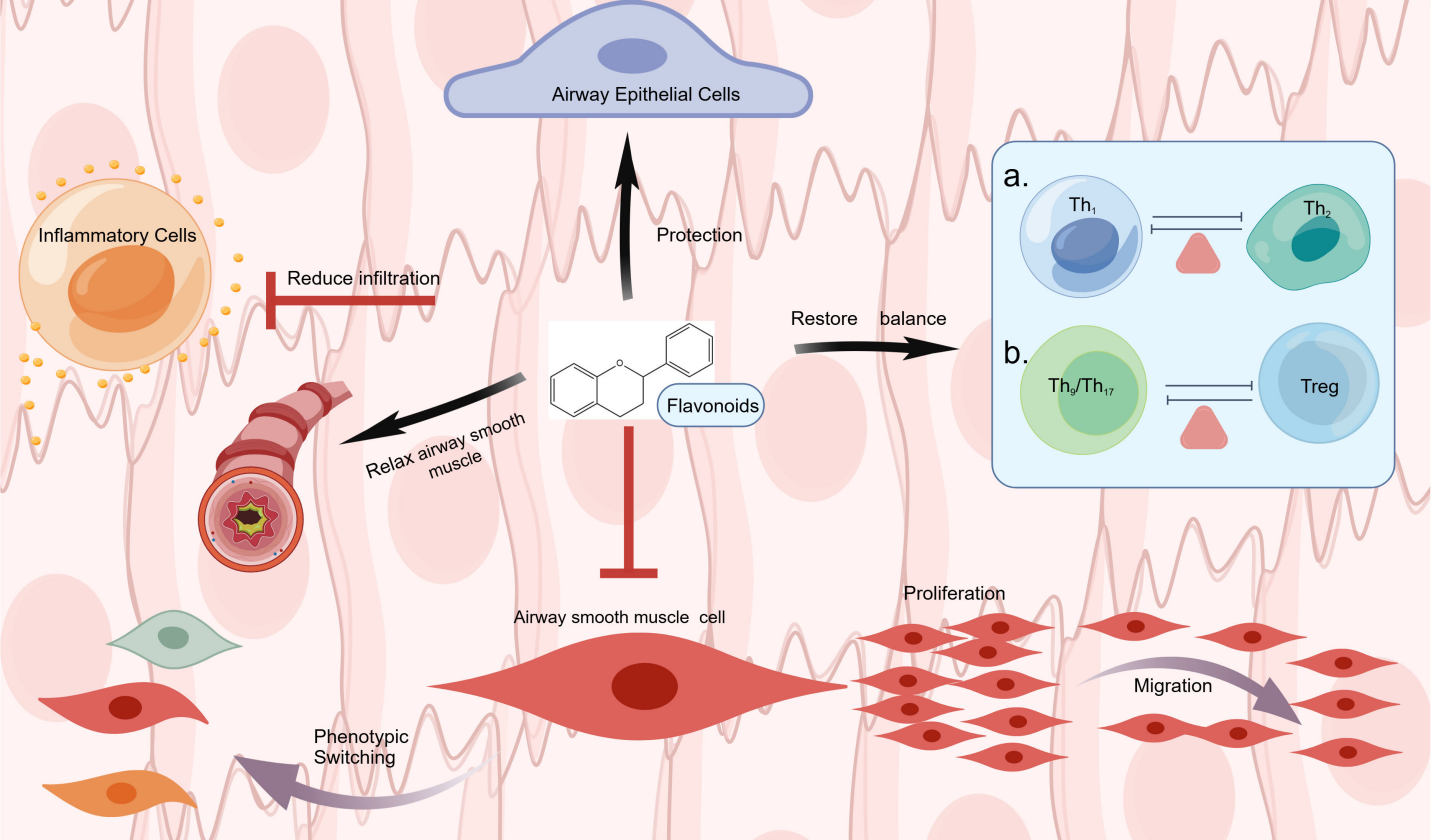
The mechanism of flavonoids against key asthmatic pathologies. Created with BioGDP.com (8).

Asthma, a chronic inflammatory airway disease affecting approximately 260 million people globally and causing an estimated 450,000 deaths annually, poses a serious threat to human health and life quality (1). It is characterized by chronic airway inflammation involving multiple cells and components, leading to airway hyperresponsiveness (AHR), narrowing, and a range of symptoms such as wheezing, breathlessness, chest tightness, and coughing (2, 3). Current mainstay pharmacotherapies, particularly beta 2 agonists and glucocorticoids, are effective for symptom control but are associated with well-documented systemic side effects and do not offer a cure (4). In this context, flavonoids, have garnered increasing scientific interest. They are renowned for their pleiotropic biological activities, including potent antioxidant, anti-inflammatory, and immunomodulatory effects (5). Recent studies continue to support an inverse association between flavonoid intake and the risk or prevalence of asthma. In a cross-sectional study of 14,520 U.S. adults, Lyu et al. found that higher total flavonoid intake was nonlinearly associated with a reduced risk of asthma, with flavanones and anthocyanidins as the major contributors (6, 7). In a cross-sectional analysis of 15,753 U.S. adults using National Health and Nutrition Examination Survey data (2007–2010 and 2017–2018), Wu et al. demonstrated that higher dietary intakes of total flavonoids, anthocyanidins, flavanones, and flavones were significantly associated with a lower prevalence of chronic respiratory diseases, including asthma, emphysema, and chronic bronchitis (7). However, there is still a lack of summary on the mechanism of flavonoids in key pathological processes of asthma. Consequently, this review summarizes the evidence on how flavonoids modulate key asthma pathologies to provide a comprehensive mechanistic basis for their therapeutic potential (**Figure 1**).

## 2 Flavonoids classification

**Figure 2:**
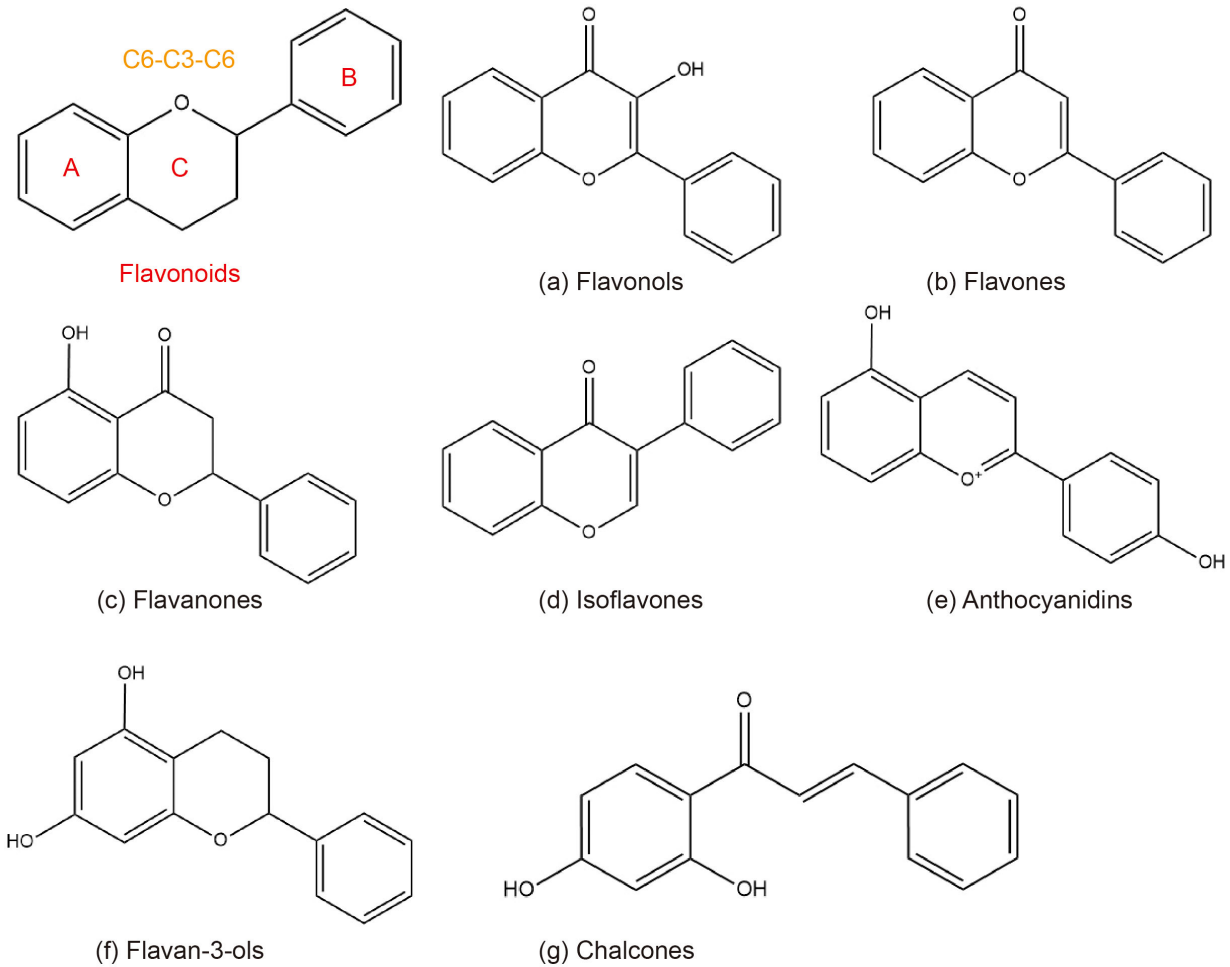
Flavonoids classification. **(a)** Flavonols, **(b)** Flavones, **(c)** Flavanones, **(d)** Isoflavones, **(e)** Anthocyanidins, **(f)** Flavan-3-ols, **(g)** Chalcones.

The fundamental architecture of flavonoids is C6–C3–C6 skeleton (labeled A, B, and C; **Figure 2**). Depending on structural differences, flavonoids are classified into seven subclasses, which include flavonols, flavones, flavanones, isoflavones, anthocyanidins, flavan-3-ols, and chalcones (**Figure 2**) (9).

### 2.1 Flavonols

Flavonols, chemically referred to as 3-hydroxyflavones, possess a distinctive structure where the A and B rings are linked by a three-carbon bridge. On the A ring, hydroxyl substituents are attached at positions 5 and 7. Common representatives of flavonols include kaempferol, quercetin, myricetin, and galangin (10, 11).

### 2.2 Flavones

Flavones, one of the largest classes of flavonoids, consists of 4H-chromen-4-one, bearing a phenyl substituent at position 2 (12). Most flavones are 7-O-glycosides. Apigenin and luteolin are two main flavones (13).

### 2.3 Flavanones

Flavanones, also termed dihydro-flavones, possess a saturated C ring. Flavanones feature a saturation of the double bond between the position C2 and C3. Among flavanones, the most representative flavanones are hesperidin and naringin, naringenin and eriodictyol (14).

### 2.4 Isoflavones

The chemical structure of isoflavones is based on the 3-phenyl chromen-4-one backbone. In soybeans, they exist as a group of 12 isomers, including genistein and daidzein. Owing to their structural resemblance to estradiol-17β and their similarity to animal estrogens, they also function as phytoestrogens. Furthermore, genistein and daidzein exhibit considerable antioxidant capacity among soy isoflavones (15).

### 2.5 Anthocyanidins

Anthocyanins are glycosides of polyhydroxy and polymethoxy derivatives. Different anthocyanins arise from variations in the number and location of hydroxyl and methoxyl substituents attached to the flavylium core. Delphinidin, cyanidin, petunidin, peonidin, malvidin, and pelargonidin represent the most commonly occurring anthocyanins (16).

### 2.6 Flavan-3-ols

Flavan-3-ols, also referred to flavanols, are characterized by the hydroxyl group bound to position 3 of the ring C. Their lack of a double bond between positions 2 and 3 differs from many other flavonoids. The hydroxyl groups which present on the A, B and C rings, differentiates them from other flavonoids. Flavan-3-ols, including catechin, gallocatechin 3-gallate, gallocatechin, epicatechin, epicatechin 3-gallate, catechin 3-gallate, and epicatechin 3-gallate, are commonly found in many fruits (17, 18).

### 2.7 Chalcones

Chalcones (1,3-diaryl-2-propen-1-ones) represent a group of open-chain natural flavonoids. They carry up to three modified or unmodified C5-, C10-, and C15-prenyl moieties on both the rings A and B. Serving as precursors for flavonoids and isoflavonoids, their structural features are easily constructed from simple aromatic compounds. Xanthohumol and isbavirachalone are two representative derivatives with abundant biological and pharmacological activity (19).

## 3 Anti-inflammatory effects of flavonoids

Airway inflammation is the most basic pathological feature of asthma (20, 21). Even patients with mild or no symptoms often show characteristic inflammatory changes in the airway mucosa and epithelium (22–24). Therefore, the elimination of airway inflammation has become the key to the treatment of asthma. Flavonoids, with their pleiotropic bioactivities, intervene at multiple levels of this inflammatory cascade, offering a multifaceted therapeutic strategy. This section outlines the anti-inflammatory mechanisms of flavonoids in the context of asthma, focusing on two key aspects: their direct modulation of inflammatory immune cells, such as eosinophils, mast cells, and neutrophils, and their protective effects on airway epithelial cells, including restoration of barrier integrity and regulation of epithelial-derived inflammatory mediators.

### 3.1 Effects on inflammatory cells

In asthma, eosinophils, neutrophils, and mast cells are key inflammatory cells, which can also mediate the production of inflammatory cytokines such as interleukin-4 (IL-4), interleukin-5 (IL-5), and tumor necrosis factor-alpha (TNF-α) in the airways of asthma patients (25, 26). These effector cells are ultimately regulated by upstream immune cells, including dendritic cells (DCs) and group 2 innate lymphoid cells (ILC2s), which shape the initiation and amplification of allergic airway inflammation (27, 28).

Quercetin, a flavonol, showed anti-inflammatory abilities in a murine model of airways allergic inflammation, where quercetin could decrease the recruitment of eosinophils to the bronchoalveolar fluid and reduced the levels of the cytokines IL-4, IL-5, IL-25 and IL-33 (29, 30). Jung et al. found that quercetin and rutin may have therapeutic effects on the late-phase response and specific airway resistance in asthma by inhibiting histamine release, PLA2 and EPO activity, as well as reducing the recruitment of neutrophils and eosinophils to the lungs (31). Wei et al. showed that baicalin can inhibit eosinophil infiltration in the bronchoalveolar lavage fluid (BALF) of allergic asthma mice, reduce the levels of inflammatory cytokines and serum IgE secretion. Furthermore, baicalin effectively alleviates the pathological changes induced by OVA in asthma and significantly inhibits the expression of thymic stromal-derived peptide (TSLP) in lung tissues (32). Jing Yao et al. found that chrysin, a flavone, not only could reduce ovalbumin (OVA)-induced increases in the number of inflammatory cells, especially eosinophils, IL-4 and IL-13 in bronchoalveolar lavage fluid and total IgE in serum, but also could upregulate the decreased interferon-γ (IFN-γ) level. Additionally, chrysin could suppress inflammatory cell infiltration, goblet cell hyperplasia and the expression of α-smooth muscle actin (α-SMA) around bronchioles. Their results indicated the promising therapeutic effect of chrysin on chronic asthma (33). Angélica Flores-Flores et al. explored the potential anti-asthmatic effect of 6-hydroxyflavone in allergic OVA-induced bronchoconstriction in a guinea pig model. 6-hydroxyflavone exhibited significant anti-asthmatic activity, as evidenced by marked bronchodilation compared to control groups. This effect was attributed to the inhibition of mast cell degranulation and/or reduction in IgE, IL-4, IL-5, and IL-13 levels (34). Genistein exerted its anti-asthma effect by stabilizing mast cells (35). Xue et al. discovered that hesperidin, neohesperidin, naringin, and narirutin are the main components of Aurantii Fructus Immaturus flavonoids (AFIFs). AFIFs can effectively inhibit allergic asthma both *in vivo* and *in vitro* by suppressing inflammatory reactions and mast cell degranulation (36). Luteolin was found to alleviate neutrophilic asthma by inhibiting IL-36γ secretion-mediated MAPK pathways, which made it potential in the treatment of neutrophilic asthma (37). Wei et al. showed that soybean isoflavones (Genistein and Daidzein) selectively inhibit the maturation of dendritic cells induced by TLR-4/LPS, downregulate the costimulatory molecules and Major Histocompatibility Complex class I molecules on the surface of human dendritic cells (DCs), thereby weakening the ability of DCs to induce T cell activation and Th_1_ cytokine secretion, and enhance the recognition and killing of DCs by Natural Killer Cells (NK cells). *In vivo*, dietary intake of isoflavones can effectively inhibit the mucosal immune response induced by nasal sensitization and reduce the production of antigen-specific antibodies. These findings revealed the potential value of Genistein and Daidzein as immunomodulators in inhibiting airway inflammation-related immune sensitization, providing experimental evidence for their application in the prevention and treatment of inflammatory diseases such as asthma (38). In an OVA-induced murine asthma model, Bao et al. reported that oral soy isoflavone for 28 days dose-dependently alleviated AHR, reduced eosinophil and neutrophil infiltration in BALF, and suppressed pulmonary mRNA expression of eotaxin, IL-4 and IL-5, while upregulating IFN-γ expression (39).

In addition to the above-mentioned effects on classical effector cells, flavonoids also modulate upstream immune populations. For instance, Liu et al. found that Formononetin improves allergic asthma by targeting JUN to inhibit type II immune responses and ILC2 migration (40). Yang et al. first demonstrated that three Glycyrrhiza uralensis flavonoids (Isoliquiritigenin, 7,4’-dihydroxyflavone and liquiritigenin) contained in Anti-asthma Herbal Medicine Intervention (ASHMI) could inhibit memory Th_2_ cells *in vitro*. 7,4’-dihydroxyflavone reduced the production of IL-4, IL-5, and cell proliferation without altering cell viability. This compound also strongly inhibited the production of the key Th_2_ cell transcription factor GATA-3. In a mouse model, 7,4’-dihydroxyflavone reduced some antigen-induced Th_2_ responses, including the production of Th_2_ cytokines and antigen-specific IgE, as well as airway inflammation, while increasing IFN-γ (41).

In addition to the classical Th_2_-driven eosinophilic asthma, flavonoids have also shown therapeutic potential in more complex asthma phenotypes involving Th17/neutrophil-driven inflammation. In a chronic obese asthma mouse model, apigenin effectively inhibits inflammatory cell proliferation, reduces levels of IL-4, IL-5, IL-13, IL-17, TNF-α, and IFN-γ, and corrects Th_17_-mediated inflammatory homeostasis disruption, with higher doses demonstrating superior efficacy. This model exhibits mixed granulocytic asthma characterized by elevated neutrophils and eosinophils, alongside a Th_17_/Th_1_/Th_2_ mixed inflammatory pattern; apigenin demonstrates potent suppression of this complex inflammation and exerts immunoregulatory effects (42). Similarly, nobiletin dose-dependently downregulates mRNA expression of Th_2_ cytokines (IL-4, IL-5, IL-13) and neutrophil-associated pro-inflammatory mediators (IL-6, IL-17a, TNF) in lung tissue of OVA-sensitized mice, while targeting both the Th_2_ response and neutrophil/IL-17A-driven pathological processes by inhibiting the STAT3 and PI3K/AKT signaling pathways, thereby providing a novel strategy for treating refractory asthma (43). Studies on sakuranetin further confirm that this compound reduces serum IgE levels, eosinophil and neutrophil counts, and Th_2_/Th_17_ cytokine levels in the lungs, and suppresses activation of ERK1/2, JNK, p38, and STAT3, thereby alleviating airway inflammation and mucus secretion (44). These findings demonstrate that flavonoids can modulate the Th_17_/IL-_17_/neutrophil axis through multi-target mechanisms, exerting anti-inflammatory and immunomodulatory effects in complex asthma phenotypes, suggesting potential clinical development value.

### 3.2 Protection and modulation of airway epithelial cells

The airway epithelium is not merely a passive barrier but an active participant in inflammation, releasing chemokines and cytokines that recruit immune cells. Flavonoids help restore epithelial integrity and function (45). Kaempferol is a natural flavonol-type flavonoid that has been isolated from citrus fruits, apples, broccoli, and so on (46). Gong et al. employed human airway epithelial BEAS-2B cells and asthma mice model to investigate the effects of kaempferol on endotoxin- or cytokine-associated airway inflammation. They found that kaempferol could suppress LPS-induced eotaxin-1 protein expression via mediating Janus kinase 2 signaling and attenuate TNFα-induced expression of epithelial intracellular cell adhesion. Additionally, kaempferol could blunt TNFα-induced airway inflammation through weakening monocyte chemoattractant protein-1 transcription via disturbing NF-κB signaling. Further animal studies showed that oral administration of kaempferol could significantly reduce the level of eotaxin-1 and eosinophil major basic protein through blocking NF-κB transactivation, that is responsible for preventing eosinophil accumulation in airway and lung tissue (47). In a recent study, kaempferol ameliorated the progression of chronic airway inflammation by decreasing the levels of IL-5, IL-13, granulocyte-macrophage colony-stimulating factor (GM-CSF), eosinophil count in bronchoalveolar lavage fluid and TGF-β1 protein level in lung tissue (48). Additionally, Caglayan Sozmen et al. reported that quercetin could reduce epithelial thickness, subepithelial smooth muscle thickness, goblet and mast cell numbers in allergic airway inflammation mice model (30). Thereby, the beneficial effects on inflammation might be related to modulating epithelium derived cytokines and epithelial apoptosis. Apigenin, a flavone, protected airway epithelial cells from apoptosis by inhibiting the MAPK pathway. It suppressed ERK, JNK, and p38 phosphorylation, upregulates Bcl−2, and downregulates Bax and cleaved caspase−3, thereby preserving epithelial integrity (49). Additionally, scutellarin, a flavone glucuronide, alleviated airway remodeling in mice and suppresses TGF-β1-induced migration and epithelial-mesenchymal transition in human bronchial epithelial cells via MAPK and Smad2/3 signaling (50).

## 4 Flavonoids targeting T cell subset balance

The pathogenesis and progression of asthma are intricately linked to dysregulated immune responses, particularly imbalances among CD4^+^ T helper (Th) cell subsets. In addition to the well-established Th_1_/Th_2_ paradigm, accumulating evidence highlights the critical roles of Th_9_, Th_17_, and regulatory T cells (Tregs) in asthmatic inflammation. A reduction in Treg numbers and a concomitant increase in Th_17_ cells represent key pathogenic features, with Treg cells maintain immune homeostasis by suppressing aberrant Th2 responses (51). Accordingly, therapeutic strategies aimed at restoring the balance among these T cell subsets have become a central focus of asthma immunotherapy.

### 4.1 Maintain the balance of Th_1_/Th_2_

The onset and progression of asthma are closely associated with dysregulated immune responses, particularly an imbalance in T helper cell subsets (52, 53). Initial CD4^+^ T cells (naive CD4^+^ T Cells) can be differentiated into a subgroup of T helper (Th) cells with diverse functions in asthma state, such as Th_1_, Th_2_, Th_9_ and Th_17_, among which the ratio of Th_1_/Th_2_ plays a dominant role for asthma (54). Th_2_ cells promote allergic inflammation by secreting IL-4 and IL-5, with IL-5 playing a critical role in eosinophil recruitment and activation, and IL-4 mediating Th_2_ polarization and IgE isotype switching (55, 56). However, Th_1_ cells have the opposite effect to Th_2_ cells. They inhibit asthma response by releasing Th_1_ cytokines such as IFN-γ to antagonize the function of Th_2_ cytokines (57, 58). At present, it is generally believed that there is a Th_1_/Th_2_ imbalance with Th_2_ dominance in asthma patients (59). T-bet and GATA-3 are two major Th-specific transcription factors, which regulate the expression of cytokine genes and play important roles in T cell differentiation (60). The change in the T-bet/GATA-3 ratio always results in the change in the Th_1_/Th_2_ balance. Therefore, the T-bet/GATA-3 ratio can serve as an indicator for assessing the balance of Th_1_/Th_2_ immune responses in asthma. Regulating the balance between Th_1_ and Th_2_ has thus emerged as a key focus in asthma immunotherapy.

Flavonoids have demonstrated significant potential in correcting this imbalance, primarily by suppressing Th_2_ responses while promoting or preserving Th_1_ activity (61). This immune-regulating effect helps balance Th_1_**_/_**Th_2_ cell responses, reduce IgE levels, and change T-bet and GATA-3 expression. Tectorigenin (a methoxyisoflavone), naringin (a flavanone glycoside), and icariin (a flavonol glucoside), could balance the Th_1_/Th_2_ levels in asthma models (62–64). They could suppress the infiltration of inflammatory cells and protect from airway inflammatory reactions and remodeling via several anti-inflammatory and immunomodulatory mechanisms, such as upregulation of the Th_1_ and control the generation of the Th_2_-type cytokines, suppressing increased IgE production, keeping the T-bet/GATA-3 ratio balance, and so on (65).

### 4.2 Maintain the balance of Th_9_/Treg cells and Th_17_/Treg cells

Besides the level varying of Th_1_/Th_2_, the imbalance between Th_9_/Th_17_ cells and forkhead box protein 3 (Foxp3)^+^ regulatory T (Treg) cells also serve as critical regulators of allergic immunity in asthma. Epimedin C, a flavonol glycoside, is a dominant component of *Herba Epimedii* with immunoregulatory activity. Muhua Huang et al. investigated the effect of epimedin C on modulating the balance between Th_9_ cells and Treg cells through inhibiting noncanonical NF-κB pathway and activating ERK and p38 MAPK. They found that epimedin C treatment could significantly increase the percentage of Treg cells and decrease the percentage of Th_9_ cells in lungs of asthmatic mice. Additionally, epimedin C decreased the IL-9 mRNA expression and IL-9 secretion, meanwhile, increased transcription factor Foxp3 mRNA expression and IL-10 secretion. The result supported the hypothesis that epimedin C regulated the balance between Th_9_ cells and Treg cells resulting in suppressing allergic airway inflammation (66).

The decrease in Treg cells and the increase in the number of Th_17_ cells in asthma patients are also important pathogenic mechanisms of asthma (59, 67–70). It was revealed that IL-6 can combine with TGF-β to promote Th_17_ differentiation and inhibit Treg cell function (71). Icariin could effectively inhibit the expression of IL-6, IL-17, TGF-β to promote FOXP3 expression and Treg cell development and differentiation, further significantly reduce Th**_17_**/Treg cell balance (72). Apigenin suppressed Th_17_ cell responses in allergic asthma. It reduced the proportion of Th_17_ cells and downregulates RORγt protein levels, accompanied by decreased IL−17A, IL−6, and TNF−α, thereby ameliorating airway inflammation (73).

## 5 Airway remodeling effects of flavonoids

Airway smooth muscle cell (ASMC) proliferation and migration are key pathological features of airway remodeling in asthma. Additionally, increasing evidence identifies phenotypic switching of airway smooth muscle cells (ASMCs) from a contractile to a synthetic state as a central driver of this process (74).

### 5.1 Inhibition of proliferation and migration

Numerous studies have demonstrated that flavonoids can inhibit the proliferation and migration of airway smooth muscle cells, contributing to preventing airway remodeling. Icariside II, a natural flavonol glycoside, attenuated eosinophil-induced airway inflammation and remodeling in asthmatic mice by inhibiting TGF-β1-induced proliferation and migration of ASMCs via inactivation of NF-κB and STAT3 signaling (75). Eupatilin, a flavone, alleviated airway remodeling by regulating the phenotypic plasticity of ASMCs, effectively suppressing TGF-β1-induced proliferative and migratory responses (76). Licochalcone A, a chalcone, inhibited VEGF-induced ASMC proliferation and induces cell cycle arrest, further supporting the anti-proliferative role of flavonoids in remodeling intervention (77). Fisetin, a flavonol, reduced ovalbumin-triggered airway remodeling by preventing phenotypic switching and inhibiting ASMC migration, potentially through the PI3K/AKT pathway (74).

### 5.2 Phenotypic switching

Increasing evidence identifies phenotypic switching of ASMCs as a central driver of airway remodeling. Recent studies have highlighted the therapeutic potential of flavonoids, in attenuating airway remodeling by directly targeting ASMC phenotypic plasticity. Fisetin has been shown to suppress ovalbumin-induced airway remodeling *in vivo*, an effect attributed to its ability to prevent ASMC phenotypic switching and preserve a contractile phenotype, thereby positioning fisetin as a promising candidate for anti-remodeling intervention (74). Similarly, procyanidin B2, flavan-3-ol dimer, mitigates asthmatic airway remodeling by inhibiting TGF-β1-induced ASMC proliferation through modulation of ROS signaling, effectively reversing the synthetic phenotypic shift (78). Likewise, eupatilin alleviates airway remodeling via regulating the phenotypic plasticity of ASMCs, further reinforcing the mechanistic link between flavonoid intervention and phenotype-driven remodeling (76).

## 6 Regulation of ASM contractility and AHR by flavonoids

ASM contractility and AHR are the proximate causes of bronchoconstriction in asthma. Accumulating evidence indicates that certain flavonoids exert acute bronchodilator effects by interfering with key contractile signaling pathways in ASM.

One of the most direct lines of evidence comes from studies on 6-hydroxyflavone. Using an *ex vivo* tracheal rat ring bioassay, Flores-Flores et al. demonstrated that 6-hydroxyflavone -induced relaxation is primarily mediated through blockade of calcium channels, as well as through the production of the relaxant second messengers’ nitric oxide (NO) and cyclic guanosine monophosphate (cGMP) (34).

Beyond calcium channel blockade, other flavonoids target distinct components of the contractile machinery. Brown and colleagues evaluated several flavonols for their ability to relax ASM and identified galangin and fisetin as potent relaxants. Both compounds significantly relaxed acetylcholine-precontracted murine tracheal rings and human ASM strips *ex vivo*, and inhaled galangin attenuated methacholine-induced increases in lung resistance *in vivo*. Mechanistically, galangin and fisetin inhibited both phosphodiesterase-4 (PDE4) and phospholipase-Cβ (PLCβ) enzymes, attenuated procontractile Gq agonist-induced intracellular calcium elevation, and reduced acetylcholine-stimulated inositol phosphate production and CPI-17 phosphorylation in human ASM cells. This dual inhibition of PLCβ and PDE4 represents a novel, β-agonist-independent mechanism for achieving ASM relaxation (79). Zhang et al. found that (-)-Epigallocatechin-3-gallate (EGCG) may inhibit airway inflammation, AHR, and mucus secretion during asthma exacerbations through the TNF-α/TNF-R1/NLRP3 signaling pathway (80). Another flavonoid, trifolirhizin isolated from Sophora flavescens, has been shown to inhibit acetylcholine-induced ASM contraction and relax pre-contracted ASM. Notably, this effect is independent of β_2_-adrenoceptors, as it was not blocked by the β-antagonist propranolol, suggesting an alternative signaling pathway distinct from conventional bronchodilators (81).Similarly, the flavonoids apigenin and luteolin, components of Dracocephalum kotschyi, produced concentration-dependent relaxation of both acetylcholine- and KCl-induced contraction in isolated rabbit tracheal rings, with effects comparable to the standard bronchodilator aminophylline (82).

IL-13 and IL-17A sensitized ASM to contractile agonists through multiple, partially distinct mechanisms. IL-13 promoted enhanced calcium signaling, upregulation of the RhoA/ROCK pathway, induction of contractile receptor expression, and activation of β1 integrins. IL-17A primarily acted via the RhoA/ROCK pathway and β1 integrin activation (83). The flavonoids fisetin and 6-hydroxyflavone effectively intervene in these pathways. Fisetin primarily suppressed the expression of IL-13, IL-17A, and other cytokines by inhibiting NF-κB activation (84), whereas 6-hydroxyflavone induced direct airway relaxation via calcium channel blockade and the NO/cGMP pathway (34). Both compounds significantly improved airway AHR in animal models, suggesting that flavonoids have the potential to ameliorate asthmatic AHR by modulating cytokine-driven pathways.

## 7 Structure-activity relationship of flavonoids for asthma

Understanding the structure-activity relationship (SAR) of flavonoids is therefore essential for identifying the key molecular features that govern their anti-inflammatory and anti-asthmatic efficacy, as well as for guiding the rational design of more potent derivatives.

The substitution pattern on the B-ring is a major determinant of anti-inflammatory potency. Silva et al. explicitly stated that “hydroxyl groups in the B ring and/or positioned as in the catechol group” are crucial for the anti-inflammatory action of flavonoids. They further noted that the “catechol group in B ring showed to be a determinant factor for the enzyme inhibition” of lipoxygenase (LOX), which is a pro-inflammatory mediator (85). Supporting this, Li et al. systematically compiled 184 natural flavonoids with anti-inflammatory activities (IC_58_ ≤ 50 μM). Among the most active compounds were luteolin and quercetin. It is well established from the basic chemistry of flavonoids that both luteolin and quercetin possess hydroxyl groups at the C3’ and C4’ positions of the B-ring (86).

Beyond B-ring features, the regulatory enzymes of the arachidonic acid cascade are essential for both inflammation and immune response. Silva et al. similarly stated that hydroxylation of A and B rings, C2-C3 unsaturation and ketone group in C4 contribute to increasing the inhibitory activity on the kinases (85). Lago et al. explained that the extended π conjugation in flavonoids (ketonic carbonyl at C-4 and C-2/C-3 double bond at ring C) stabilizes the intermediate radical species involved in inflammation, thereby providing the mechanistic basis for this requirement (87).

However, in the context of *in vivo* anti-asthmatic activity, certain glycosides maintain or even enhance efficacy. Specifically, Chung et al. compared kaempferol (KF, aglycone) and its glycosylated derivative kaempferol-3-O-rhamnoside (K-3-rh) in an OVA-induced asthma mouse model and found that K-3-rh maintained anti-inflammatory and anti-asthmatic effects comparable to KF. Notably, K-3-rh exhibited an even stronger inhibitory effect on serum total IgE production than KF (88).

## 8 Comparative analyses of flavonoid effects versus approved asthma medications

To compare the therapeutic potential of flavonoids with current asthma drug treatments, we evaluated the effects of flavonoids against those of approved anti-asthma medications, including dexamethasone, salbutamol, and Th_2_-targeted biologics. After intervention with Formononetin or DEX, the levels of total IgE and OVA-IgE in serum were significantly decreased. Furthermore, compared to the model group, the levels of inflammatory mediators IL-4, IL-9, and IL-13 in the BALF of mice in the Formononetin or DEX treatment groups were significantly reduced. The results indicated that, compared to the model group, Formononetin and DEX treatments decreased the proportion of ILC2s in lung tissue by approximately 2.85% and 3.11%, respectively (40).

Similar comparative evidence was also obtained in a guinea pig allergic asthma model. Quercetin and rutin significantly inhibited sRaw and leukocyte recruitment. The inhibitory levels of quercetin and rutin in late-acting response (LAR) were similar to that of DEX, and in immediate-acting response (IAR) were similar to that of salbutamol. Therefore, quercetin and rutin can be used for the treatment of IAR and LAR in asthma (31).

In addition to these traditional small molecule drugs, the therapeutic characteristics of flavonoids can also be compared with modern biotherapies currently used in clinical practice. Unlike the limited efficacy of biologics targeting Th_2_ targets (such as omalizumab or dupilumab) in neutrophilic asthma, nobiletin targets two aspects of allergic inflammation: (1) regulating Th_2_ cytokine activity through STAT3-mediated GATA3 expression inhibition, and (2) regulating neutrophil/IL-17A-driven pathological processes through PI3K/AKT inhibition. This dual action disrupts the self-amplifying neutrophil extracellular trap (NET)-Th_2_ cycle, making nobiletin a promising adjuvant therapy for mixed-granulocyte infiltration asthma resistant to biologics (43).

## 9 Clinical translation challenges and pharmacokinetic optimization of flavonoids

A systematic review of 15 randomized controlled trials on flavonoids in allergic diseases identified 3 asthma-specific trials involving 990 participants. While 80% of the studies reported some clinical benefits, the remaining 20% found no significant improvement in symptom scores or lung function. According to Grading of Recommendations, Assessment, Development, and Evaluation (GRADE) assessment, the overall quality of evidence was low to moderate (89).

The gap between preclinical promise and clinical reality is perhaps best illustrated by a large multicenter randomized controlled trial of soy isoflavone supplementation in asthma. Smith et al. enrolled patients with poorly controlled asthma and administered soy isoflavones daily for a period of months. Despite achieving plasma genistein levels sufficient to inhibit eosinophil leukotriene synthesis *in vitro*, the supplement failed to improve Forced Expiratory Volume in the first second (FEV1), symptoms, or quality of life compared with placebo. These negative findings highlight that robust preclinical mechanistic data and epidemiological associations do not guarantee therapeutic efficacy in well-controlled clinical trials, underscoring the critical importance of rigorous clinical evaluation in flavonoid research (90, 91).

In addition to the inconsistent clinical evidence, the poor pharmacokinetic profiles of many flavonoids represent another major barrier to clinical translation. Formononetin (FMN) has demonstrated promising anti-inflammatory, antioxidant, and antifibrotic effects in preclinical studies. However, its clinical translation is severely constrained by poor solubility, rapid metabolism, and low oral bioavailability. To overcome these pharmacokinetic limitations, advanced delivery systems, including albumin-based nanoparticles (FMN@BSA) and PLGA-encapsulated formulations, have been developed to enhance stability, bioavailability, and targeted delivery. These nanoparticle systems have shown superior pharmacological efficacy in animal models, with inhalable carriers offering the potential for direct lung targeting in asthma and COPD, while sustained-release formulations may benefit chronic conditions such as pulmonary arterial hypertension and pulmonary fibrosis. Given their biocompatibility, biodegradability, and capacity for site-specific deposition, albumin nanoparticles and polymer-based carriers represent promising future strategies for pulmonary drug delivery (91).

## 10 Conclusions

Asthma is a complex chronic respiratory disease driven by persistent airway inflammation, dysregulated T cell immunity, and progressive structural remodeling. Although current pharmacotherapies, particularly glucocorticoids, effectively alleviate symptoms, they fail to reverse airway remodeling and are often accompanied by systemic side effects. In this context, flavonoids, naturally occurring polyphenolic compounds with broad bioactivity, have emerged as promising complementary candidates for asthma management. This review consolidates the mechanistic evidence supporting the anti-asthmatic potential of flavonoids (**Table 1**). At the inflammatory level, flavonoids suppress key pro-inflammatory signaling pathways (e.g., NF-κB, MAPK, PI3K/AKT). They also attenuate eosinophilic and neutrophilic inflammation by reducing inflammatory cell recruitment, inhibiting mast cell degranulation, and protecting airway epithelial integrity. In parallel, flavonoids restore immune homeostasis by rebalancing T helper cell subsets: they rectify the Th1/Th2 imbalance, promote Treg differentiation, and suppress pathogenic Th9 and Th17 responses through transcriptional regulation of T-bet, GATA-3, Foxp3, and related cytokines. Furthermore, flavonoids could improve airway remodeling, a pathological feature poorly addressed by current therapies by inhibiting aberrant airway smooth muscle cell proliferation and migration, and by modulating ASMC phenotypic switching from a synthetic to a contractile state. These effects are mediated through interference with TGF-β1, VEGF, and ROS signaling cascades. Flavonoids counteracted ASM hypercontractility and AHR via multiple signaling pathways, ranging from calcium channel blockade and NO/cGMP production to PLCβ/PDE4 inhibition and suppression of IL-13/IL-17A-mediated RhoA/ROCK and β1 integrin activation, highlighting their potential as multi-target bronchodilators for asthma therapy. The anti-asthmatic SAR of flavonoids indicated that the catechol group on the B-ring, C2-C3 double bond, C4 carbonyl, and appropriate hydroxylation patterns are critical for activity, while the role of glycosylation is context-dependent, sometimes preserving or even enhancing efficacy against asthma-related endpoints such as IgE production. The current understanding of the structure-activity relationships of flavonoids specifically for asthma remains somewhat incomplete. Nevertheless, the existing findings provide valuable insights and a solid foundation for future medicinal chemistry efforts aimed at developing flavonoid-based anti-asthmatic agents.

**Table 1:** Key pathological processes targeted by flavonoids in asthma.

Pathological process	Representative flavonoids	Model used	Evidence level	References
Anti-inflammatory effects	Kaempferol	BEAS-2B cells; BALB/c mice	in vitro; in vivo	(46–48)
	Chrysin	BALB/c mice	in vivo	(33)
	Luteolin	BEAS-2B cells; C57BL/6 mice	in vitro; in vivo	(37)
	Soy isoflavones (genistein, daidzein)	Human monocyte-derived DCs; BALB/c mice	in vitro; in vivo	(38, 39)
	Soy isoflavones (genistein, daidzein, and glycitein)	386 patients	clinical	(90)
T-cell subset balance	Tectorigenin	BALB/c mice	in vivo	(62)
	Naringin	BALB/c mice	in vivo	(63)
	Apigenin	BALB/c mice	in vivo	(73)
Airway remodeling inhibition	Icariside II	BALB/c mice; ASMCs	in vivo; in vitro	(75)
	Fisetin	BALB/c mice; ASMCs	in vivo; in vitro	(74)
	Scutellarin	BALB/c mice; 16HBE	in vivo; in vitro	(50)
ASM contractility and AHR	Trifolirhizin	BALB/c mice, tracheal rings	ex vivo	(81)
	Galangin, Fisetin	A/J mice, Murine tracheal rings; Human ASM strips;	ex vivo; in vivo	(79)
	6-Hydroxyflavone	Rat tracheal rings; guinea pigs	ex vivo; in vivo	(34)

To bridge the gap between preclinical findings and potential clinical application, we estimated the human equivalent dose (HED) using allometric scaling based on body surface area (92). The effective mouse doses (~100 mg/kg) correspond to HEDs of approximately 8.1 mg/kg for a 60 kg adult (~486 mg/day). Notably, this upper estimate (486 mg/day) is close to the 500 mg/day threshold associated with the greatest reduction in all-cause and cardiovascular mortality in a large Danish cohort study (93). Moreover, a recent randomized controlled trial demonstrated that 500 mg/day quercetin safely reduced intrahepatic lipid content in patients with NAFLD without adverse effects (94). Given that 500 mg of total flavonoids can be obtained from a diet rich in fruits, vegetables, tea, and berries, the preclinical doses tested in this study are not only pharmacologically relevant but also clinically achievable. These findings support the potential translation of flavonoid-based strategies into dietary recommendations or adjunctive therapies.

It should be acknowledged that this review is not a systematic meta-analysis, and therefore, no formal quality assessment was conducted on the included studies. Furthermore, we recognize the potential for publication bias, as studies reporting positive results are more likely to be published, which may lead to an overestimation of the anti-asthma efficacy of flavonoids. In the future, well-designed, large-scale clinical trials are necessary to validate these preliminary observations.
